# Literary Retrospect

**Published:** 1863-10

**Authors:** 


### LITERARY RETROSPECT.

By the Public Health Act of 1858, the sanitary powers previously exercised by the General Board of Health were transferred to the Privy Council. The ordinary duties thus imposed upon the Privy Council (as contradistinguished from the responsibilities which rest upon it in times of formidable epidemics) were the regulation of public vaccination, and the investigation, as might be needed, of matters concerning the public health in any place or places, and into the observance of the regulations and directions issued by them under the Act. It was provided also that the medical officer of the Privy Council—to which post Mr. Simon, the medical officer of the General Board of Health, was appointed on the dissolution of the Board—should make an annual report to the Council of the proceedings which had been taken under the Act during the previous twelve months; and that from time to time he should report also on subjects touching the public health, or on such matters as might be referred to him.

The fifth of the annual reports which the Act thus enjoins to be made has recently been published. It is a document of unusual importance, whether it be regarded in relation to the subjects with which it is specially occupied, or in reference to previous reports and ultimate results which are likely to be obtained by the system of procedure adopted by the department. The Report is occupied with a further account of the inquiry instituted four years ago by the Privy Council into the state of public vaccination, and which has now extended over half the kingdom. It contains also certain investigations into industrial diseases—to wit, an inquiry, conducted by Dr. Guy, on the occupations which have to do with arsenical green; and an inquiry, by Dr. Bristowe, on the occupations which have to do with phosphorus. In addition, the Report contains a detailed account, of great value, of an investigation, by Professor Gamgee, into the diseases of live-stock, in their relation to the public supplies of meat and milk. Further, there are reports of inquiries, by Dr. Buchanan, into the health of the distressed operatives in Lancashire, and on the health of the girls in the sewing-schools at Preston; also, by Dr. Ed. Smith, on the nourishment of the distressed cotton-workers. Some of these investigations had been made public before the publication of Mr. Simon's report, and all possess peculiar value.

Comparing this report with previous reports, it is apparent that while the medical department of the Privy Council ably provides for the practical needs of the moment, under the guidance of Mr. Simon, a mass of evidence is being collected calculated to form a solid foundation for an accurate and continuous knowledge of the sanitary condition of the population throughout the entire kingdom, and in its different districts. If this surmise be correct, we may hope that the time is not far distant when that great desideratum will be supplied—authoritative reports, at stated intervals, on the condition of public health in every portion of the country.

Are there malarious poisons? This question Dr. Herbert Barker has attempted to solve in an Essay* to which the Fothergillian prize of the Medical Society of London was awarded. The work is of great interest. It contains much ingenious research and experiment, and not the least of its merits is its suggestiveness.

A welcome addition to our library shelves is Dr. Abbotts Smith's† Treatise on Human Entozoa. It is a good practical treatise, well adapted for the busy practitioner, and we commend it to our readers.

---

* *On Malaria and Miasmata, and their Influence in the Production of Typhus and Typhoid Fevers, Cholera, and the Exanthemata.* By T. Herbert Barker, M.D. Lond. 8vo. pp. 251. 1862.

† *On Human Entozoa, comprising the different Species of Worms found in the Intestines and other Parts of the Human Body, and the Pathology and Treatment of the various Affections produced by their presence. To which is added a Glossary of the principal Terms employed.* By Wm. Abbotts Smith, M.D., M.R.C.P., Senior Assistant-Physician to the Metropolitan Free Hospital, &c. 8vo. Lond. pp. 245.

Dr. Waters is very honourably known as the author of an Essay on the Anatomy of the Human Lung, to which the Medical Society of London awarded the Fothergillian gold medal. His anatomical investigations have naturally led him to a consideration of the pathological condition of the pulmonary tissue. In a treatise of great merit, he examines the nature, pathology, and treatment of emphysema of the lungs.* Dr. Waters maintains that "the disease in its severer forms is of a constitutional nature; that one of its most important features, and perhaps the primary step in it, is a malnutrition of the pulmonary tissue, causing its degeneration, and giving rise to all the structural changes he has previously described." The main principles of treatment would, therefore, be such as guide us in the treatment of diseases attended with degeneration, as, for example, Bright's disease and fatty degeneration of the heart. Dr. Waters gives the following summary of the circumstances which induce him to believe that emphysema is the result of some degenerative process:—"(1.) The higher degree of development which the disease often reaches, without any previous history of violent or long-standing cough, either in connexion with bronchitis, hooping-cough, or any similar affection. (2.) The frequency with which the disease attacks the whole of both lungs, and the uniformly equal character of the morbid changes often observed throughout all parts of the lungs. (3). The hereditary nature of the disease, as shown by the observations I have alluded to. (4). The manner in which the disease is influenced by certain remedial measures which are known to act beneficially on other diseases attended with degeneration of tissue." The detailed account of the treatment which Dr. Waters advocates in the disease is comprehensive and excellent.

In 1853 Dr. Fisher, of Boston (U. S.), published a memoir on *a cephalic bellows sound*, and averred that "auscultation may be as useful in the symptomatology of affections of the brain as in those of the chest, and may furnish a pathognomonic sign of these diseases." He stated that he had detected an abnormal bruit as one of the symptoms of chronic hydrocephalus, cerebral congestion, either simple or resulting from disordered dentition or hooping cough, acute inflammation of the encephalon or its membranes, abscess of the brain and induration of that organ. Subsequently Dr. Whitney, a colleague of Dr. Fisher, stated that the symptomatological value of the cephalic *souffle* was even greater than Dr. Fisher's researches had shown. He had found it diagnostic of scirrhous transformation of the cerebellum and mechanical compression of the brain, in addition to the maladies named by Dr. Fisher. He stated, moreover, that he had detected a *cerebral ægophony* in hydrocephalus, a *bruit* similar to the *catarrhal fremitus* in aneurism of the arteries at the base of the brain, and finally he points to the existence of a *cephalic souffle* in *anæmia of the brain* and also in chlorosis.

---

* *Researches on the Nature, Pathology, and Treatment of Emphysema of the Lungs, and its Relations with other Diseases of the Chest.* By A. T. H. Waters, M.D., M.R.C.P. 8vo. London. pp. 114. 1862.

The observations of Dr. Fisher and Dr. Whitney did not attract much attention in Europe. Although apparently founded on a large number of facts, they did not inspire confidence. Recently, however, M. Henri Roger has subjected the whole question of a cephalic *souffle* and its semeiological value to a systematic research, and a translation of his highly interesting memoir on auscultation of the head has been published by Dr. Meadows.* M. Roger's researches, while furnishing no confirmation of the extravagant value attached to cerebral auscultation by the two American physicians, nevertheless lead to certain positive results of considerable value. The conclusions to which he has been led by his investigations are thus summed up:—

"1. The existence of a cephalic *souffle* in the chloro-anæmia of very young subjects. In this it is of very frequent occurrence, while it is quite exceptional in affections of the brain. 2. The nature of this *souffle*, which is always a sound connected with an alteration of the blood, an inorganic not an organic sound. 3. The frequency of chloro-anæmia during the first year of life, and at the epoch of dentition. 4. The frequency of anæmia in pertussis, equally misunderstood. 5. The possibility of the early recognition of the changed condition of the blood by means of auscultation of the cranium. This enables it to be promptly met, which is of importance in very early life, when every cause of enfeeblement of the economy may lead to (especially where there is predisposition) general tuberculosis. 6. The frequency, if not constancy, of the cephalic *souffle*, as well as carotidean sounds, in rickets. 7. The demonstration, by this *souffle* and its characters, of the nature of rickets, which should not be regarded as a disease limited to the osseous system, but as a change in the condition of the blood, as a disease implicating the whole economy. 8. The demonstration, by means of exact figures, of the epoch when the fontanelles commence closing (at ten months in a fourth of the subjects), and of that at which such closure should be complete (between two and three years in almost all the cases); this fact is not without its pathological and juridical importance. On the one hand a delay in the closure indicates a retardation of the general ossification, and announces the imminence of rickets, or the commencement of hydrocephalus; and, on the other hand, a premature closure of the sutures and fontanelles indicates the possibility of a microcephalon with consecutive idiotcy. The determination of the condition of the fontanelles at a given period of early infancy may aid the medical jurist in approximatively determining the adult age of a young subject, or in resolving a question of identity."

The Professor of Materia Medica in the University of Edinburgh seems to have the rare and happy faculty of instilling into his students

---

* *Clinical Researches on Auscultation of the Head.* By M. Henri Roger, Member of the Imperial Academy of Medicine, Physician to the Hospital for Children. Translated from the French by Alfred Meadows, M.D., M.R.C.P., formerly Assist.-Physician for Diseases of Women and Children at King's College Hospital. London, 8vo. pp. 47. 1863.

much of that love of research, that impartial enthusiasm and philosophic spirit, which have imparted such high value to his own writings, and have built up the lofty reputation which he now enjoys. To say nothing of the distinction which many of his former pupils have attained in those departments of science with which the name of Christison is so indissolubly connected, we need only refer at present, in support of our statement, to two admirable monographs, which may almost be said to have emanated from his class-room—the treatise of Professor Fleming on the Aconitum Napellus, which at once assumed a proud place in medical literature, and is still regarded as a model for imitation in such investigations, and a paper on the Physostigma Venenosum, or Calabar Bean, by Dr. Thomas R. Fraser,* which is at present before us. Both these papers were presented as *theses* at the period of graduation, and both were awarded a gold medal by the Senatus Academicus. The latter has just been reprinted from a contemporary, and contains matter of great interest and importance. We recommend it to the attention of those of the profession who are not already familiar with Dr. Fraser's researches. To Dr. Fraser undoubtedly belongs the credit, not merely of having instituted a series of most curious and accurate experiments, but of having arrived at results of great practical utility. The action of this agent upon the iris, which will always be associated with Dr. Fraser's name, is of itself a discovery of the greatest consequence, while its influence upon other portions of the animal economy appears to indicate that it will yet be beneficially employed in various diseases.

The publication of a third edition, revised and enlarged, of Mr. Jabez Hogg's Manual of Ophthalmoscopic Surgery is the most satisfactory testimony to the merits of the work, and the estimation in which it is held.†

Mr. J. B. Curgeneven has published Jenner's account of the origin of the Vaccine Inoculation,‡ from an original copy in his possession. Let our readers hasten to secure this most brief but most instructive history. The republication is exceedingly well-timed.

It is refreshing at a time when lunacy has become the cat's-paw, so to speak, of the "sensation" writers of the present day—writers who find in the sad malady, as popularly understood and commonly misconceived, a ready resource for the exercise of their extravagant vagaries—to meet with a novelist who has the boldness to study the disease from nature, and the power of adapting it to the uses of legitimate fiction, without distorting the truth or pandering to popular prejudices. *Shirley Hall Asylum* § professes to be the memoirs of a mono-

---

* *On the Character, Actions, and Therapeutic Uses of the Ordeal Bean of Calabar.* By Thomas R. Fraser, M.D. Edinburgh. 1863. 8vo, pp. 44.

† *A Manual of Ophthalmoscopic Surgery; being a Practical Treatise on the Use of the Ophthalmoscope in Diseases of the Eye.* By Jabez Hogg. 3rd edit. revised and enlarged. 8vo, Lond. pp. 296. 1863.

‡ *On the Origin of the Vaccine Inoculation.* By Edward Jenner, M.D., F.R.S. London. 4to. pp. 8. (Elfick, 22, Leinster-terrace, W.) 1863.

§ *Shirley Hall Asylum; or, the Memoirs of a Monomaniac.* Lond. Sm. 8vo, pp. 393. 1863.

maniac, and to describe the mode of life, as well as to recount the histories of some of the inmates of a private asylum. The work is written with a Defoe-like graphicness, which is as attractive as it is novel in these days of spasmodic literature.

Dr. S. Haughton, F.R.S., propounds, with a brevity probably unique in recondite physiological literature, the Outlines of a New Theory of Muscular Action.*

To America belongs the unhappy honour of having published the first text-book on military hygiene in the English language. There has recently issued from the Philadelphia press an able work on this subject, by Dr. Wm. A. Hammond, Surgeon-General U.S. Army. The interest of this work is not circumscribed by its special application, and we recommend it to the attention of all practitioners.†

An able essay on *Drink Craving*, by Dr. Robert Bird, of the Bengal Medical Establishment, is an interesting contribution to our knowledge of the subject, and is of special value in connexion with the hygiene of the Indian army—a question now largely occupying the public attention.

There are also lying on our table a continuation of Mr. Lockhart Clarke's invaluable Researches on the Microscopic Anatomy of the Nervous Centres, reprinted from the *Philosophical Transactions;* and an investigation made by him into the Condition of the Nervous Centres in a Case of Wasting Palsy, reprinted from the *Archives of Medicine.* Mr. Clarke's researches into the intimate pathology of the nervous centres are opening out an entirely new and most fruitful field of knowledge. We would note also reprints of the following papers:—"On the Convulsive Diseases of Infants," by Dr. Ballard; "The Connexion between Tuberculosis and Insanity," by Dr. Clouston; also, several papers on Cancer of the Female Sexual Organs, Cases in Obstetric Medicine, and of Uterine Affections, by Dr. Tanner. Finally, we would direct attention to a singularly interesting paper on "Various Superstitions in the North-west Highlands and Islands of Scotland, especially in Relation to Lunacy," by Dr. Arthur Mitchell, Deputy Commissioner of Lunacy in Scotland, reprinted from the *Proceedings of the Scottish Society of Antiquaries.*

---

* *Outlines of a New Theory of Muscular Action.* A Thesis read for the Degree of M.D. before the University of Dublin, Dec. 17, 1862. By the Rev. Samuel Haughton, M.D., F.R.S. Sm. 8vo. Lond. 1863.

† *A Treatise on Hygiène, with special reference to the Military Service.* By Wm. B. Hammond, M.D. Sq. 8vo, pp. 604. Philadelphia, 1863.

